# Green Synthesis of Nitrogen-Doped Carbon Dots from *Pueraria* Residues for Use as a Sensitive Fluorescent Probe for Sensing Cr(VI) in Water

**DOI:** 10.3390/s25175554

**Published:** 2025-09-05

**Authors:** Ziyuan Zheng, Zhengwei Zhou

**Affiliations:** School of Environmental Science and Engineering, Changzhou University, Changzhou 213164, China; s23030857077@smail.cczu.edu.cn

**Keywords:** carbon dots, *Pueraria* residues, fluorescent probe, hexavalent chromium (Cr(VI)), density functional theory (DFT)

## Abstract

In this study, blue fluorescence carbon dots of high quantum yield (42.96%) were successfully synthesized via a one-step hydrothermal method using *Pueraria* residues as the precursor and urea as the nitrogen source. The preparation process was simple, was environmentally friendly, and did not use toxic chemicals, with the resulting nitrogen-doped *Pueraria* carbon dots (N-PCDs) exhibiting excellent dispersibility, regular morphology and stable fluorescence performance. Moreover, fluorescence quenching could be induced through electron transfer between N-PCDs and hexavalent chromium (Cr(VI)) in water, which enabled the application of N-PCDs as a fluorescent probe for sensing Cr(VI) in water, with a limit of detection (LOD) and limit of quantitation (LOQ) of 0.078 μM and 0.26 μM, respectively. The effectiveness of the proposed fluorescent probe was also validated in various water matrices, achieving stable recovery rates ranging from 98.7% to 101.5%. Furthermore, experimental investigations and theoretical calculations through density functional theory (DFT) confirmed that the underlying reaction mechanism was photoinduced electron transfer (PET). Above all, this study not only demonstrated the potential of N-PCDs as sensitive probes to sense toxic elements in the environment, but also promotes the green and scalable production of high-value carbon-based products from waste biomass.

## 1. Introduction

Environmental contamination by chromium species predominantly originates from leather products, industrial pigments, substandard cosmetic ingredients, metallic electroplating, and rubber manufacturing [1,2]. Exposure to chromium species, especially hexavalent chromium (Cr(VI)), can induce severe health risks including dermatoses, hepatic cirrhosis, hemolytic anemia, and elevated carcinogenic potential [3,4]. Current detection methods for heavy metals demonstrate several critical limitations, including reliance on sophisticated instrumentation, strict requirements for sample pretreatment and prohibitive procedural development costs [5]. Hence, it is of practical significance to develop Cr(VI) detection methods with rapid response as well as enhanced selectivity and sensitivity.

As a group of zero-dimensional carbon-based nanomaterials, carbon dots (CDs) have been widely applied in photocatalysis, chemosensing, lasers, bioimaging and diagnosis due to their exceptional stability, biocompatibility and optoelectronic properties [6,7,8,9,10]. Currently, multiple synthesis methods exist for such unique nanomaterials, including microwave irradiation [11], hydrothermal treatment [12], laser ablation [13], ultrasonication [14], solvothermal processing [15], the ball-milling method [16] and chemical oxidation [17]. However, escalating demand for high-performance CDs has revealed the inherent limitations of conventional synthetic routes, particularly referring to costly and toxic raw materials and low yields of CDs [18]. Therefore, the development of eco-friendly synthetic routes utilizing non-toxic and sustainable carbon sources has attracted much attention. Recent research has progressed in preparing biomass-derived CDs, taking advantage of natural, non-toxic, and cost-effective lignocellulosic precursors [19,20,21]. For instance, Hu et al. utilized flax straw as the carbon source and successfully synthesized CDs with a quantum yield (QY) of 20.7%, and the prepared CDs could detect Co(II) and Cr(VI) in water samples with limits of detection (LODs) of 0.38 μM and 0.19 μM, respectively [22]. In another study, Bhatt et al. prepared CDs with a LOD of 0.087 μM for Cr(VI) detection using tulsi leaves as the precursor; the QY was only 3.06% [23]. Nevertheless, despite the developments in waste-derived CDs for Cr(VI) determination, challenges remain due to several issues such as the complicated preparation process, relatively low QYs of CDs, and low selectivity in sensing toxic chemicals. Therefore, it is still of vital significance to explore new waste biomass as a precursor for CD production and develop a rapid and efficient Cr(VI) sensing technique.

In China, approximately 1 million tons of puerarin powder are produced every year, which then generate 10 million tons of *Pueraria* residue [24]. These agricultural byproducts are currently subject to inefficient management, as only a small proportion are reutilized as animal feed while the majority are discarded through field deposition or incineration, leading to considerable environmental pollution and resource wastage [25]. On the other hand, comprehensive compositional analysis reveals that *Pueraria* residues contain approximately 41% cellulose, 16% hemicellulose, and 25% lignin [26], indicating its viability as a potential precursor for CD production. However, to the best of our knowledge, there has been no public report till now focusing on the utilization of *Pueraria* residues for CD production.

Herein, we established a green synthesis pathway for CDs via a one-step hydrothermal method using urea and *Pueraria* residues as precursors, yielding blue fluorescent nitrogen-doped *Pueraria* CDs (N-PCDs) with excitation-dependent emission characteristics, which were then employed as a fluorescent sensor for the detection of aqueous Cr(VI). The synthesized N-PCDs were comprehensively characterized to investigate their morphological, optical, and structural properties. The changes in fluorescence intensity upon the addition of varying concentrations of Cr(VI) ions were also systematically studied. Moreover, the proposed analytical method was applied for Cr(VI) detection in various water matrices to validate applicability in a real-world scenario. Finally, the detection mechanism was validated through density functional theory (DFT) calculations of orbital energy and fluorescence lifetime analysis.

## 2. Materials and Methods

### 2.1. Materials

The *Pueraria* residues were collected from Jurong City, Jiangsu Province, China. All other chemicals and reagents were purchased from SinoPharm Co. Ltd. (Shanghai, China), and used without further treatment. Distilled water was used in the experiments.

### 2.2. Characterization Techniques

Transmission electron microscopy (TEM) and high-resolution transmission electron microscopy (HR-TEM) images were obtained using a Tecnai F20 TEM (FEI, Eindhoven, The Netherlands) and a JEM-2100plus (JEOL, Tokyo, Japan) TEM, respectively. UV–visible spectra were recorded on a UV-3000PC spectrophotometer (Mapada, Shanghai, China). Fluorescence spectra were obtained on a Cary Eclipse Fluorescence Spectrophotometer (Agilent, Santa Clara, CA, USA). The attenuated total reflection–Fourier-transform infrared spectra (ATR-FTIR) were recorded on a Hyperion3000 Vertex70 FTIR spectrophotometer (Bruker, Bremen, Germany). X-ray diffraction (XRD) patterns were determined on a SmartLab SE X-ray diffractometer (Rigaku, Tokyo, Japan) and recorded at a scanning rate of 5° min^−1^ with 2*θ* value ranging from 10 to 80°. X-ray photoelectron spectra (XPS) were determined on a Scientific K-Alpha spectrometer (Thermo, Waltham, MA, USA). The fluorescence lifetime was recorded on a FLS1000 fluorescence spectrometer (Edinburgh, Livingston, UK).

### 2.3. Synthesis of N-PCDs

**Scheme 1:**
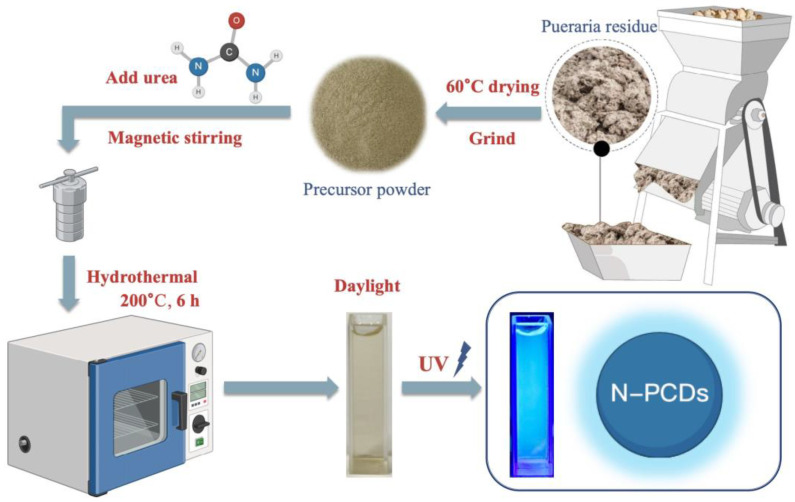
Schematic representation of synthesis of N-PCDs.

N-PCDs were synthesized via the hydrothermal method, and the preparation procedure is shown in Scheme 1. The detailed steps are as follows: A certain weight of dry *Pueraria* residues were collected and further dried at 60 °C for 24 h to remove moisture. The thoroughly dried residues were finely ground, and 1 g of the resulting powder was dispersed in 10 mL of distilled water. The mixture was magnetically stirred at 1000 rpm for 20 h to ensure complete homogenization. Subsequently, 10 mL of the dispersion was mixed with 10 mL of a 1 M CO(NH_2_)_2_ aqueous solution and stirred for 15 min. The resulting mixture was transferred to a 50 mL Teflon-lined autoclave and maintained at 200 °C for 6 h. After cooling to room temperature, the supernatant was centrifuged and then filtered through a 0.22 µm membrane filter. Afterwards, N-PCDs were obtained through freeze-drying in a vacuum freeze dryer and then redispersed in water with a concentration of 10 mg/L for further Cr(VI) detection.

### 2.4. Quantum Yield Calculations

The fluorescence quantum yield (QY) of the synthesized N-PCDs was determined using the reference method. Quinine sulfate in a 0.1 M H_2_SO_4_ solution, with a known QY of 54% under excitation at 365 nm, was employed as the reference standard [27]. The concentration of quinine sulfate was adjusted to match its absorption intensity with that of the synthesized N-PCDs, ensuring that the absorbance of both the reference solution and the N-PCDs suspension was maintained at approximately 0.05. The fluorescence quantum yield of the synthesized N-PCDs was subsequently calculated using the following formula:

$${QY}_{N-PCDs}={QY}_{R}\times \frac{F}{F_{R}}\times \frac{A_{R}}{A}\times \frac{\eta^{2}}{\eta_{R}^{2}}$$

Here, N-PCDs and R represent carbon dots and reference points, respectively. The parameters QY, F, A, and *η* represent quantum yield (quinine sulfate = 0.54 under excitation at 365 nm), integrated fluorescence intensity, absorbance of the sample at the excitation wavelength, and refractive index of the solvent medium (*η* = 1.33 for both quinine sulfate and N-PCDs).

### 2.5. Fluorometric Detection of Cr(VI)

The selectivity of the synthesized N-PCDs was investigated using fluorescence spectroscopy by examining the effects of various metal ions (Al^3+^, Zn^2+^, Cr^3+^, Mg^2+^, Ni^2+^, Cu^2+^, Ca^2+^, Mn^2+^, Pb^2+^, Co^2+^, Hg^2+^, Fe^3+^ and Na^+^), anions (CO_3_^2−^, PO_4_^3−^, SO_4_^2−^, CH_3_COO^−^ and HPO_4_^2−^) and organic compounds (glucose and phenol) on the fluorescence intensity of the N-PCDs. Stock solutions of these ions were prepared at concentrations of 1 M, respectively. A 5 μL aliquot of the N-PCDs solution was diluted with distilled water in a cuvette to a final volume of 4 mL. The fluorescence spectrum of the resulting solution was recorded at an excitation wavelength of 340 nm and an emission wavelength of 425 nm, and the fluorescence intensity at 340 nm excitation was designated as *F_0_*. Subsequently, the diluted N-PCDs solution was mixed with 2 mL of each ion solution, and the photoluminescence (PL) intensity of the resultant mixture at 340 nm excitation was measured and denoted as *F*. Additionally, a series of Cr(VI) solutions with concentrations ranging from 0 to 500 μM were added to 5 μL of the N-PCDs solution, after which the total volume was adjusted to 4 mL with distilled water. This procedure was used to evaluate the sensitivity and anti-interference capability of the synthesized N-PCDs toward Cr(VI) ions, following the same protocol as the selectivity experiment.

### 2.6. Detection in Real-Water Samples

To investigate the environmental applicability of synthesized N-PCDs, real-water samples including river, lake and tap water samples were collected, filtered using 0.22 μm pore-sized membranes, and then spiked with standard Cr(VI) solutions to make testing samples of various Cr(VI) concentrations. The recovery rates of Cr(VI) in each matrix were then determined using the fluorescence detection method described earlier. Under identical experimental conditions, the relative standard deviation (RSD) was assessed by conducting three replicate experiments.

### 2.7. Computational Details

All the coordination configuration calculations were performed via the first-principles density of functional theory [28]. The C-material model consisted of 21 hydrogen (H) atoms, 58 carbon (C) atoms, 2 nitrogen (N) atoms, and 18 oxygen (O) atoms. The chromate ion (CrO_4_^2−^) model consisted of 4 oxygen (O) atoms and 1 chromium (Cr) atom. The computational tasks were executed using the DMol3 module within Materials Studio 2019 (BIOVIA, San Diego, CA, USA). Both platforms were grounded in DFT and employed the ultrasoft pseudopotential (USP) method. For the parameter settings, a coarse precision was selected. Specifically, the plane-wave cut-off energy was set at 381 eV, and the energy variation per atom during constant iteration was maintained below 5.0 × 10^−5^ eV. The force exerted on each atom was constrained to no more than 0.1 eV/Å, while the maximum ion displacement was limited to 0.005 Å. The exchange-correlation energy function was modeled using the Perdew–Burke–Ernzerhof (PBE) version of the generalized gradient approximation (GGA). The interaction potential between ions and electrons was characterized by the USP method. The binding energies (E_b_) were determined through the formula E_b_ = E_N-PCDs&Cr(VI)_ − E_N-PCDs_ − E_Cr(VI)_.

## 3. Results and Discussion

### 3.1. Morphology and Structural Characteristics of N-PCDs

**Figure 1:**
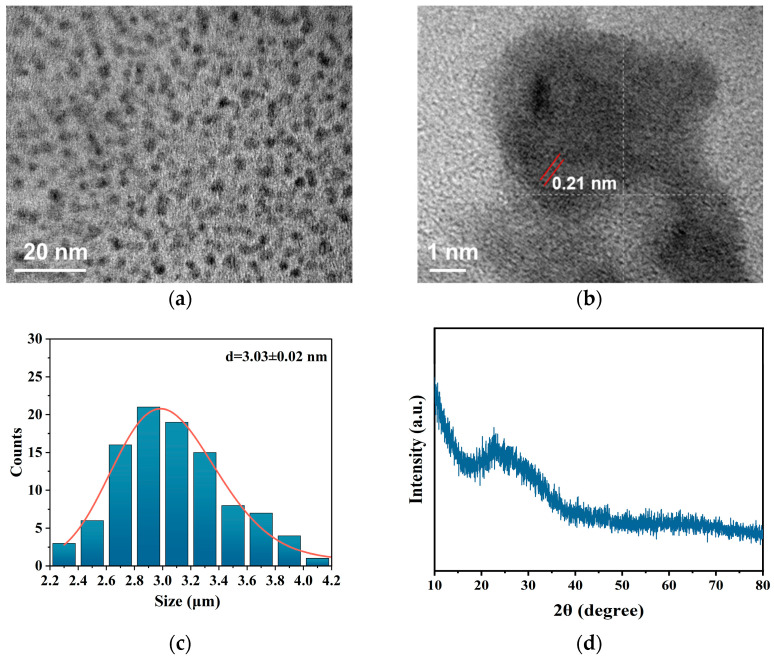
(**a**) TEM image of N-PCDs; (**b**) HRTEM image of N-PCDs; (**c**) particle size distribution histogram; (**d**) XRD pattern of N-PCDs.

The morphology and size of the prepared N-PCDs were characterized by TEM and HRTEM. The TEM image of N-PCDs (Figure 1a) shows a homogeneous dispersion of quasi-spherical nanoparticles with diameters ranging from 2.2 to 4.2 nm (Figure 1c). Meanwhile, lattice spacing of 0.21 nm was observed in the HRTEM image of N-PCDs (Figure 1b). The XRD pattern of N-PCDs (Figure 1d) exhibited a peak at 2θ of 22.8°, confirming the presence of disordered carbon atoms distributed in the (002) plane of graphitic carbon [29].

**Figure 2:**
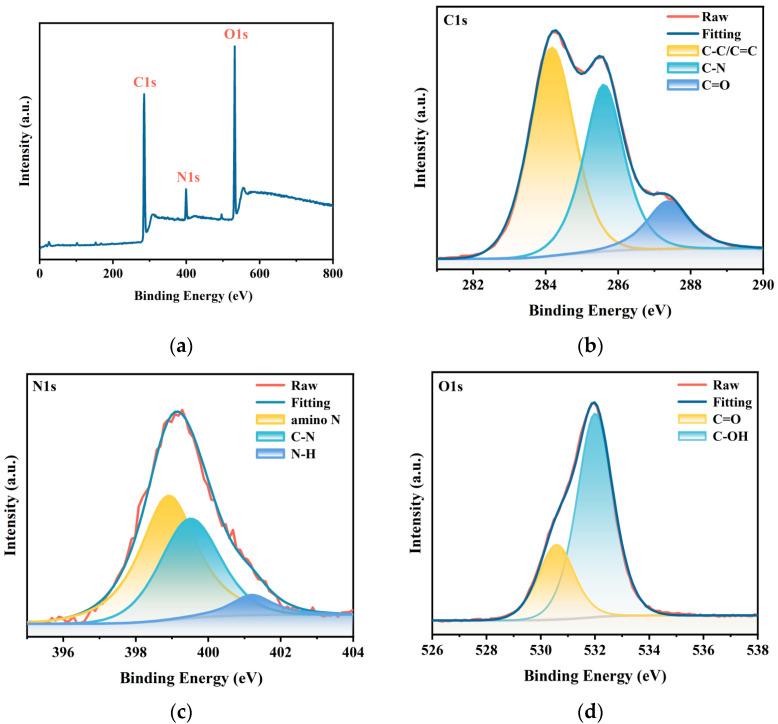
(**a**) XPS of N-PCDs. The high-resolution (**b**) C1s XPS (**c**) N1s XPS and (**d**) O1s XPS of spectra of N-PCDs, respectively.

Through the analysis of the chemical structure and elemental composition of the samples used for preparing N-PCDs, the surface functional groups of N-PCDs were further investigated using XPS. The XPS (Figure 2a) exhibited three distinct peaks at 285 eV, 399 eV, and 352 eV, corresponding to C, N, and O elements with atomic ratios of 59.74%, 8.58%, and 31.69%, respectively. The high-resolution XPS of each element revealed that the C1s spectrum (Figure 2b) could be deconvoluted into three peaks located at 284.2 eV, 285.6 eV, and 287.37 eV, which were associated with C–C/C=C, C–N, and C=O groups, respectively [30,31]. The high-resolution N1s spectrum of N-PCDs (Figure 2c) showed three peaks at 398.9 eV (amino N), 399.5 eV (C–N), and 401.2 eV (N–H) [32]. Additionally, the O1s XPS spectrum confirmed the presence of characteristic peaks at 530.6 eV (C=O) and 532.0 eV (C–OH/C–O–C) [33]. Therefore, the existence of these functional groups on the surface of N-PCDs contributed to their excellent dispersibility in water, thereby enhancing the solubility of N-PCDs in aqueous solutions.

**Figure 3:**
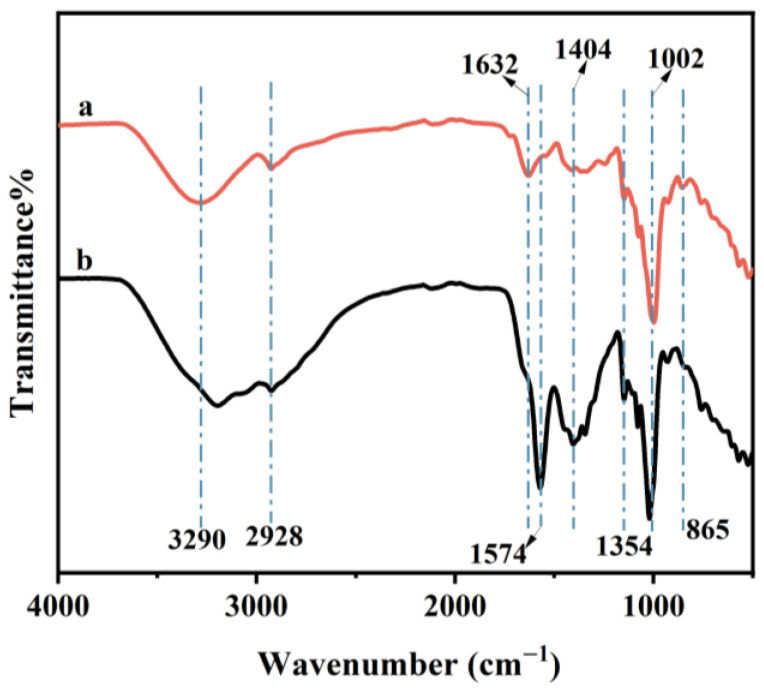
ATR-FTIR spectra of *Pueraria* residues (curve a in red) and N-PCDs (curve b in black).

The representative ATR-FTIR spectra of *Pueraria* residues and N-PCDs were comparatively analyzed (Figure 3). The absorption peak near 3290 cm^−1^ in *Pueraria* residues corresponded to the O–H stretching vibration of hydroxyl groups in cellulose molecules, indicating the hydrophilic nature of the precursor [34]. For N-PCDs, the absorption peak near 3190 cm^−1^ was attributed to the overlapping stretching vibrations of –OH and N–H bonds, suggesting the presence of –NH_2_ groups on the material’s surface after the reaction [32]. Both samples exhibited a minor peak at approximately 2928 cm^−1^, which corresponded to the asymmetric stretching vibration of the C–H group [35]. The absorption band at 1632 cm^−1^ was assigned to the stretching vibration of O–H bonds in absorbed water within the fibrous structure [24]. A notable change occurred at the peak near 1574 cm^−1^, which was associated with the stretching vibration of C=C bonds [36]. This shift might indicate structural disruption of the original fibrous framework during the reaction process. For N-PCDs, characteristic peaks at 1404 cm^−1^ and 1346 cm^−1^ were attributed to the stretching vibration of C–N bonds and the bending vibration of N–H bonds, respectively [37,38]. The peak observed at 1022 cm^−1^ corresponded to the C–O–C stretching vibration, indicating the presence of ether or glycosidic linkages [39]. Additionally, the peak at 865 cm^−1^ was assigned to the β-glycosidic bond in the glucose ring of cellulose, suggesting that some structural features were retained [40]. These results indicated that during the reaction, cellulose and hemicellulose in *Pueraria* residues were partially degraded, while the surface of the synthesized N-PCDs became enriched with oxygen-containing functional groups, thereby enhancing their hydrophilicity. This observation was consistent with the high oxygen content revealed by XPS analysis.

### 3.2. Optical Properties of N-PCDs

**Figure 4:**
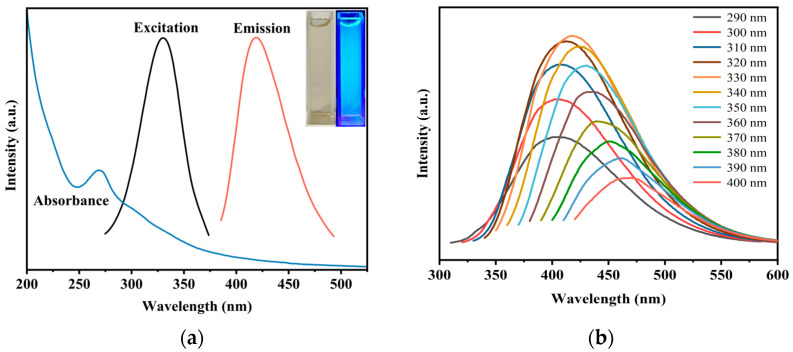
(**a**) Absorption, excitation and emission spectra of N-PCDs; (**b**) fluorescence emission spectra with different excitation wavelengths.

The fluorescence emission characteristics of the synthesized N-PCDs were investigated using UV–Vis spectroscopy and fluorescence spectroscopy. As shown in the UV absorption spectrum (Figure 4a), under daylight, the synthesized N-PCDs solution appeared yellowish-brown; under UV light, it exhibited bright blue fluorescence. In addition, a strong absorption peak near 270 nm was observed, which can be attributed to the π-π* transition of the *sp^2^*-hybridized C=C bonds in the carbon core [41]. It was also indicated that the optimal excitation and emission wavelengths for N-PCDs were 330 nm and 420 nm, respectively. Meanwhile, the fluorescence emission spectra in Figure 4b demonstrated the significant excitation-dependent behavior of N-PCDs within the excitation wavelength range of 290–400 nm. Moreover, the highest fluorescence intensity was observed at an excitation wavelength of 340 nm, leading to the selection of 340 nm as the optimal excitation wavelength. And the QY of N-PCDs (Ex = 340 nm) was then determined to be 42.96% by applying quinine sulfate in a 0.1 M H_2_SO_4_ solution as the reference (QY = 0.54%). The detailed test data is shown in Table S1.

**Figure 5:**
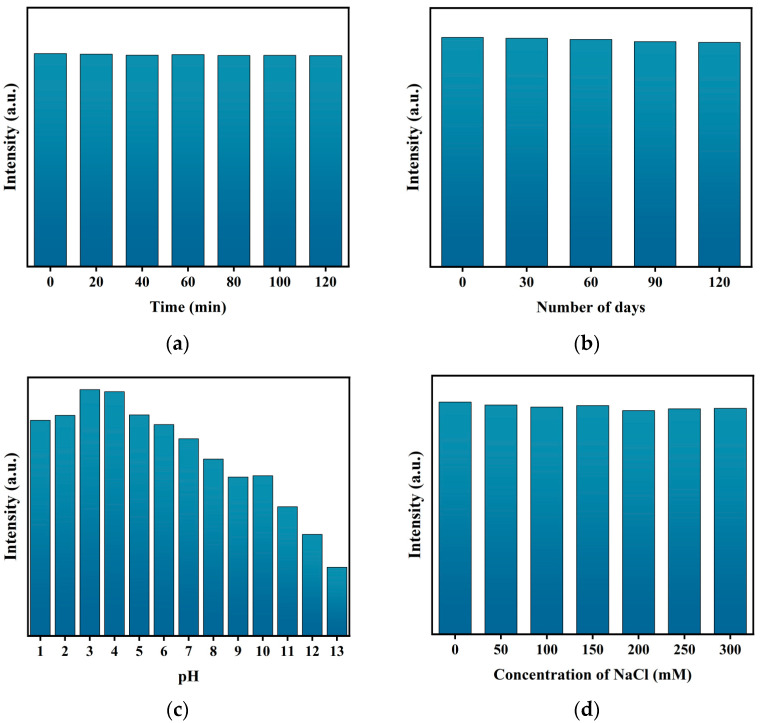
(**a**) Effect of UV light exposure duration for 120 min on the luminescence intensity of N-PCDs. (**b**) The fluorescence intensity as a function of time. (**c**) The fluorescence intensity of N-PCDs with different pH. (**d**) Effect of NaCl concentration on fluorescence intensity of N-PCDs.

The optical stability of CDs is a critical factor for their practical applications, and the fluorescence stability of the synthesized N-PCDs was systematically investigated under various conditions, including exposure time, storage duration, solution pH, and different concentrations of NaCl solution. As shown in Figure 5a, the fluorescence intensity of the N-PCDs remained stable under continuous UV irradiation (excitation/emission wavelengths: 340 nm/420 nm) for 120 min. Furthermore, the fluorescence intensity remained consistent even after 100 days, as shown in Figure 5b, indicating excellent anti-photobleaching properties of the prepared N-PCDs. In terms of the pH influence, when the solution pH was raised from 1 to 13, the fluorescence intensity demonstrated an initial increasing and then decreasing trend, with a peak at pH 3 (Figure 5c). This behavior might be attributed to the protonation and deprotonation of functional groups on the surface of the N-PCDs, which significantly influenced the fluorescence intensity. Though pH 3–4 was considered as the optimal solution pH for achieving high-sensitivity sensing performance, the sensing of Cr(VI) by N-PCDs was performed without specific pH adjustment to simplify the sensing procedure as much possible, with a mixed solution under alkaline conditions (pH: 8.0–8.2). The relatively weak fluorescence intensity under alkaline conditions might result from the rich carboxyl and hydroxyl groups on the surface of the carbon dots, leading to aggregation-induced quenching [42]. As illustrated in Figure 5d, the fluorescence intensity of N-PCDs exhibited no significant changes across different NaCl concentrations, suggesting that the surface functional groups were non-ionizable and possessed high optical stability under high-ionic-strength conditions.

### 3.3. Detection of Cr(VI)

**Figure 6:**
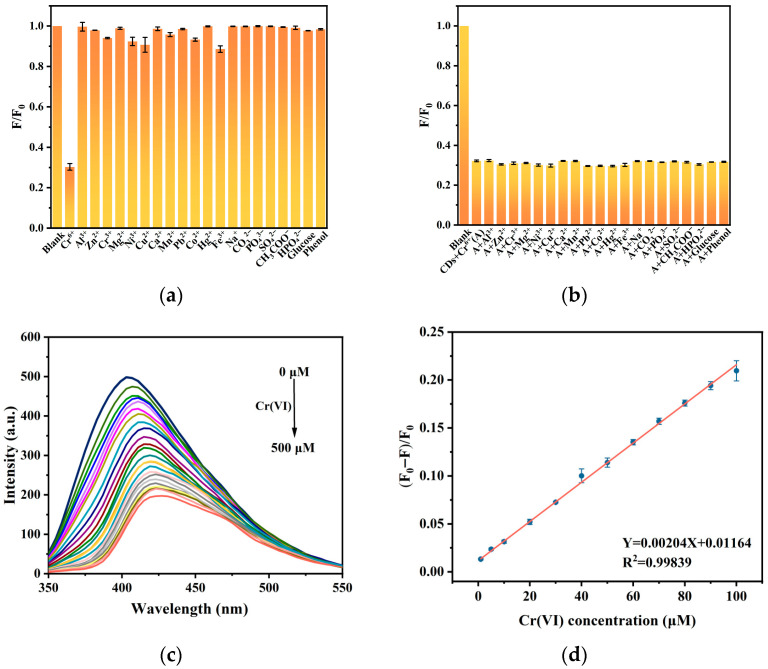
(**a**) Relative fluorescence intensity of N-PCD aqueous solution with different chemicals excited at 330 nm. (**b**) Influence of foreign chemicals on the fluorescent signal in the presence of coexisting Cr(VI). (**c**) The fluorescence intensity of N-PCDs with different Cr(VI) concentrations (0–500 μM) excited at 330 nm. (**d**) The linear dependence of (*F*_0_ − *F*)/*F*_0_ on the Cr(VI) concentration (0–100 μM).

The selectivity of the synthesized N-PCDs for Cr(VI) is shown in Figure 6a. The results demonstrated that the fluorescence intensity of N-PCDs was only significantly quenched by Cr(VI), whereas the quenching effects of other ions and organic compounds were either very weak or negligible. This phenomenon proved the superior selectivity of N-PCDs for Cr(VI). Subsequently, an interference resistance experiment for Cr(VI) detection using N-PCDs was conducted with the aforementioned chemicals. The results, as illustrated in Figure 6b, also revealed that only Cr(VI) effectively quenched the fluorescence intensity of N-PCDs, while the tested chemicals had little impact on their luminescence. These findings confirmed that the synthesized N-PCDs possessed excellent selectivity and sensitivity for Cr(VI).

The detection range and LOD were determined by monitoring the fluorescence intensity of N-PCDs as a function of Cr(VI) concentration. As shown in Figure 6c, the fluorescence intensity of N-PCDs gradually decreased with increasing Cr(VI) concentration (0–500 μM) in the aqueous solution. The relationship between the *F*_0_ − *F*/*F*_0_ value and Cr(VI) concentration is presented in Figure S1, where *F*_0_ and *F* represent the fluorescence intensities in the absence and presence of Cr(VI), respectively. At lower concentrations (0–100 μM) (Figure 6d), a strong linear relationship was observed with a correlation coefficient (R^2^) of 0.9984. The linear equation could be expressed as (*F*_0_ − *F*)/*F*_0_ = 0.00204*x* + 0.01164, where *x* denotes the Cr(VI) concentration. Based on the formula LOD = 3S/σ and limit of quantitation (LOQ) = 10S/σ, the LOD and LOQ of the N-PCDs were calculated to be 0.078 μM and 0.26 μM, respectively. Here, σ represents the generalized standard deviation of the fluorescence intensity from nine blank N-PCD solution samples, and S corresponds to the slope of the linear relationship between *F*_0_ − *F*/*F*_0_ and Cr(VI) concentration. Table S2 compares the results obtained in this study with those from the literature, and it was clear that the N-PCDs obtained in this study not only demonstrated high QY (42.96%) but also exhibited comparable performance in sensing Cr(VI) from water with the LOD of 0.078 µM. In addition, the World Health Organization (WHO) has established a provisional guideline value for total chromium intake in humans at 0.05 mg/L (0.96 μM) [32]. Consequently, the detection sensitivity of Cr(VI) using the synthesized N-PCDs fully satisfied the WHO requirements for Cr(VI) detection in liquid samples.

### 3.4. Quantitative Detection of Cr(VI) Spiked in Real-Water Samples

The practical application of N-PCDs in detecting Cr(VI) present in various water matrices was further investigated. Different concentrations of Cr(VI) ions (25 μM, 50 μM, and 100 μM) were spiked, respectively, into tap, lake, and river water samples, and the recovery rates for each concentration are summarized in Table 1. Specifically, the recovery rates of N-PCDs for Cr(VI) detection were calculated to range from 98.69% to 101.46%, with a relative standard deviation (RSD) of less than 2.12%. These results indicate the high accuracy and reliability of the proposed fluorescence probe for Cr(VI) detection in environmental water samples.

### 3.5. Detection Mechanism of N-PCDs

**Figure 7:**
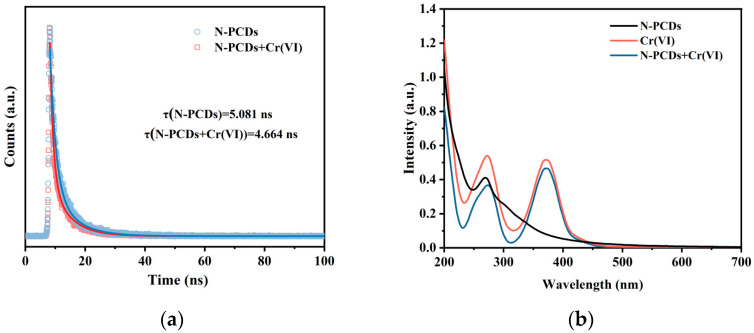
(**a**) Lifetime decay curves of N-PCDs and N-PCDs + Cr(VI). (**b**) UV–vis absorption spectra of N-PCDs, Cr(VI) and N-PCDs + Cr(VI) systems.

The quenching mechanisms of CDs usually include static quenching (SQE), dynamic quenching (DQE), fluorescence resonance energy transfer (FRET), dark resonance energy transfer (DRET), photoinduced electron transfer (PET) and inner filter effect (IFE) [43,44]. In this study, UV–Vis absorption spectroscopy and fluorescence lifetime decay analysis were utilized to investigate the quenching mechanism between N-PCDs and Cr(VI). Fluorescence lifetime experiments were performed to elucidate the mode of lifetime decay. As depicted in Figure 7a, the fluorescence decay lifetime of N-PCDs was 5.081 ns initially, and it then decreased to 4.664 ns upon the addition of Cr(VI) solution. This significant reduction in fluorescence lifetime suggested that the quenching process was dynamic rather than static. Furthermore, Figure 7b presents the UV–Vis absorption spectra of N-PCDs, Cr(VI), and their mixture (N-PCDs + Cr(VI)), in which the UV–Vis absorption spectrum of Cr(VI) (red line) exhibited two characteristic absorption peaks at 272 nm and 372 nm. Notably, the UV–Vis absorption spectrum of N-PCDs remained largely unchanged after the addition of Cr(VI), indicating that the quenching mechanism did not involve the formation of new substances. Since dynamic quenching primarily affected the excited state of the fluorophore without altering its absorption properties, these findings confirmed again that the quenching of N-PCDs by Cr(VI) was rather a dynamic process.

**Figure 8:**
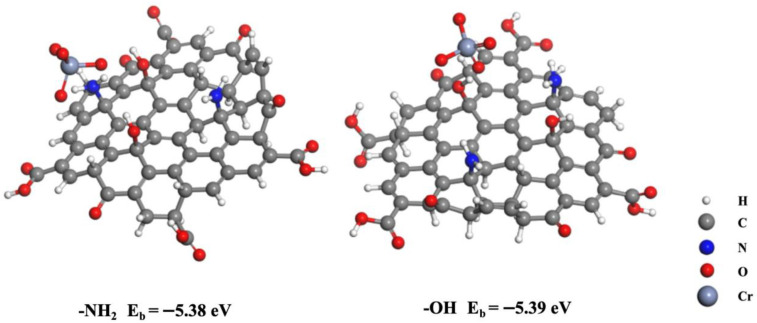
Schematic diagram of different binding sites between Cr(VI) and N-PCDs and their binding energies.

To gain deeper insights into the reaction mechanism, density functional theory (DFT) calculations were employed. Figure S2 illustrates the optimized geometric structure of N-PCDs. In the reaction solution, Cr(VI) predominantly existed in the form of chromate ions. Subsequently, the electrostatic potential (ESP) was utilized to intuitively depict the external charge distribution of N-PCDs and Cr(VI) (Figure S3), revealing the possible adsorption sites for Cr(VI). It was observed that the amino (H) and hydroxyl (H) groups exhibited positive electrostatic potential (blue), whereas the oxygen atoms of chromate ions displayed negative electrostatic potential (red). Based on these findings, it was hypothesized that the amino and hydroxyl groups served as the most probable binding sites for Cr(VI). Calculations indicated that the binding energies between chromate ions and the amino and hydroxyl groups were both high and comparable, at −5.38 eV and −5.39 eV, respectively (Figure 8). This binding might be attributed to charge transfer occurring between the two species.

**Figure 9:**
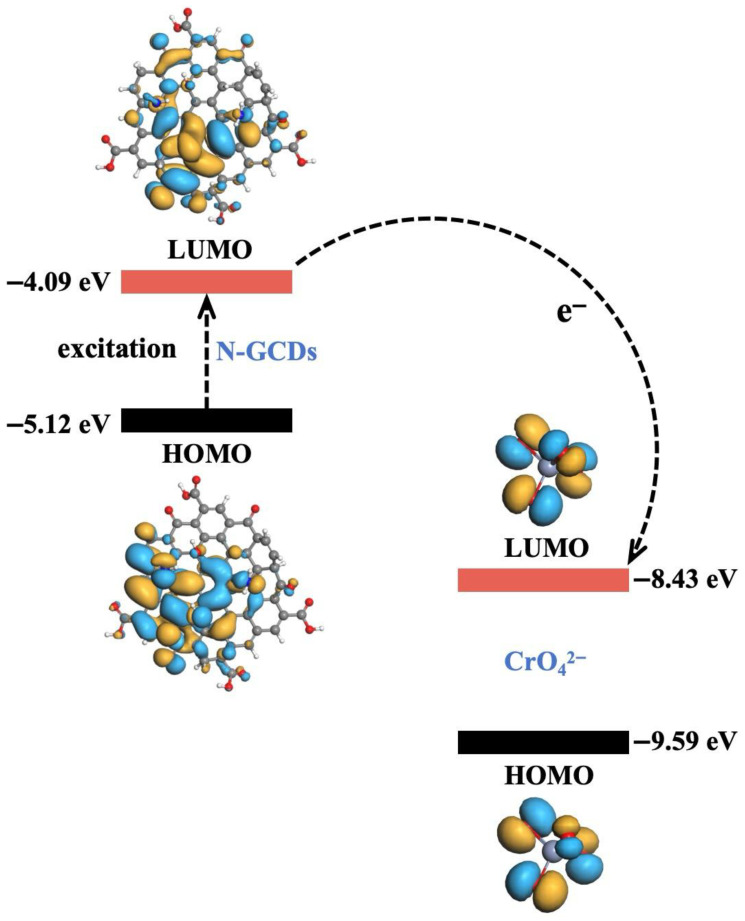
HOMO and LUMO of N-PCDs and Cr(VI) along with their energies in eV and schematic diagram of the charge transfer from N-PCDs to Cr(VI).

The detailed electron transfer mechanism is illustrated in Figure 9. Firstly, the highest occupied molecular orbital (HOMO) and the lowest unoccupied molecular orbital (LUMO) of N-PCDs were calculated as −5.12 eV and −4.09 eV, respectively. The HOMO corresponds to a π orbital, while the LUMO corresponds to a π* orbital. Then, the morphology of N-PCDs-Cr(VI) complexes and the quantified charge transfer during the reaction were simulated using Mulliken charge analysis. By comparing the Mulliken charges of chromate ions before and after binding to N-PCDs, it was observed that the Mulliken charge at the binding site significantly increased upon N-PCD binding, while the Mulliken charge of chromate ions decreased significantly (Figure S4), suggesting the acceptance of electrons from N-PCDs by Cr(VI). The electron transfer from N-PCDs to Cr(VI) induced a substantial potential drop and resulted in fluorescence quenching [45]. Based on the aforementioned analysis, it could be concluded that the quenching process of this reaction was dynamic. Moreover, the energy gap between the LUMO of N-PCDs and the HOMO of the quencher indicated that the quenching mechanism followed the PET mode [46]. Since PET involves excited-state electron transfer between the fluorophore and the quencher, this effectively explains the observed decay in the fluorescence lifetime of N-PCDs, as mentioned in Figure 7a.

## 4. Conclusions

A simple and environmentally friendly one-step hydrothermal method of high QY was developed for the synthesis of N-PCDs, using *Pueraria* residue as the precursor and urea as a green nitrogen source. A sensitive and selective fluorescence probe for Cr(VI) detection in various water matrices was proposed based on the dynamic quenching process between the prepared N-PCDs and Cr(VI), with a LOD of 0.078 μM. Theoretical calculations confirmed that PET fitted the fluorescence quenching mechanism very well. Therefore, N-PCDs derived from abundant waste *Pueraria* residues not only hold significant environmental appeal but also provide an innovative strategy for the resourceful recycling of agricultural waste.

## Figures and Tables

**Table 1:** Detection of Cr(VI) in different water samples.

Sample	Spiked (μM)	Found Conc. (μM)	%Recovery	%RSD (*n* = 3)
Tap water	25	24.93	99.73	1.66
	50	50.04	100.09	2.03
	100	100.02	100.02	0.60
Pond water	25	24.67	98.69	1.16
	50	50.73	101.46	0.31
	100	100.01	100.01	0.57
River water	25	25.32	101.27	1.57
	50	49.96	99.92	2.12
	100	100.18	100.18	1.04

RSD: Relative standard deviation.

## Data Availability

The data presented in this study are available on request from the corresponding author.

## Supplementary Materials

The following supporting information can be downloaded at: https://www.mdpi.com/article/10.3390/s25175554/s1, Figure S1. The dependence of (F_0_-F)/F_0_ on the Cr(VI) concentration (0~500 μM). Table S1. Determination of the fluorescence quantum yield of N-PCDs by referencing quinine sulfate, utilizing integrated emission intensity and absorbance at 330 nm. Figure S2. Optimized geometry of CDs showing top view and side view. Figure S3. Electrostatic potential distribution diagrams of Cr(VI) and N-PCDs. Figure S4. (a) Mulliken charge distribution map of Cr(VI) and N-PCDs. (b) The distribution map of the binding charge of Cr(VI) and N-PCDs at the -NH2 site. (c)The distribution map of the binding charge of Cr(VI) and N-PCDs at the -OH site.
