# Supplementary material for: Green Synthesis of Nitrogen-Doped Carbon Dots from Pueraria Residues for Use as a Sensitive Fluorescent Probe for Sensing Cr(VI) in Water
Source: Sensors (Basel). 2025 Sep 5;25(17):5554. doi: 10.3390/s25175554 (PMC12431478; doi:10.3390/s25175554)
Supplement: Supplementary file 1 [file sensors-25-05554-s001.zip › sensors-3804347-supplementary.pdf]

Green Synthesis of Nitrogen-Doped Carbon Dots from *Pueraria* Residues for Use as a Sensitive Fluorescent Probe for Sensing Cr(VI) in Water

Ziyuan Zheng and Zhengwei Zhou *

School of Environmental Science and Engineering, Changzhou University, Changzhou 213164, China; s23030857077@smail.cczu.edu.cn
* Correspondence: zwzhou@cczu.edu.cn

Supplementary Information

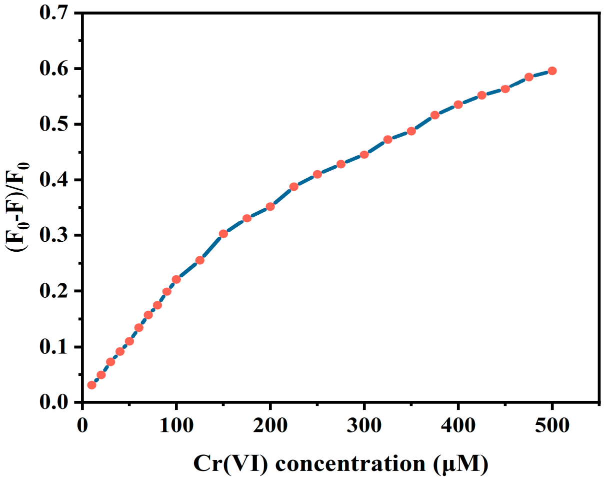

Figure S1. The dependence of $(F_0-F)/F_0$ on the Cr(VI) concentration (0~500 μM).

Table S1. Determination of the fluorescence quantum yield of N-PCDs by referencing quinine sulfate, utilizing integrated emission intensity and absorbance at 330 nm.

Sample	Integrated Intensity at 330 nm	Absorbance at 330 nm	Quantum Yield (%)
Quinine Sulphate (Reference)	4.9701	0.06	54
N-PCDs	3.9538	0.06	42.96

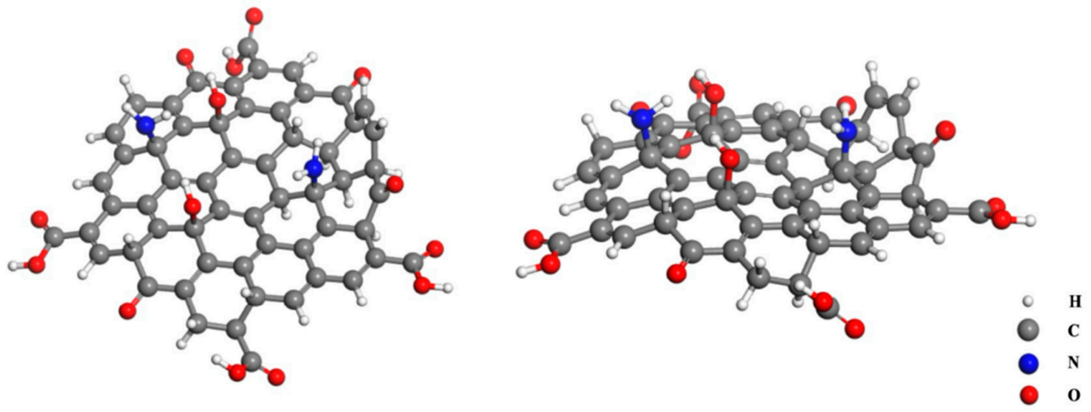

Figure S2. Optimized geometry of CDs showing top view and side view.

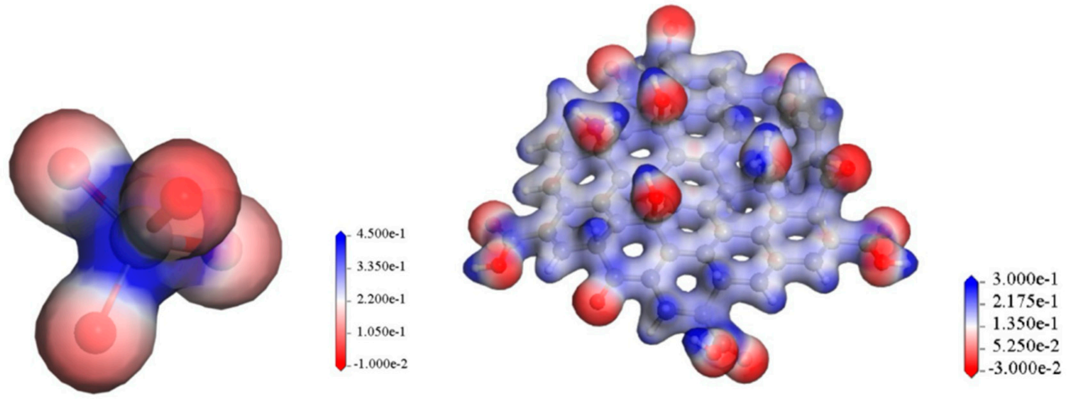

Figure S3. Electrostatic potential distribution diagrams of Cr(VI) and N-PCDs.

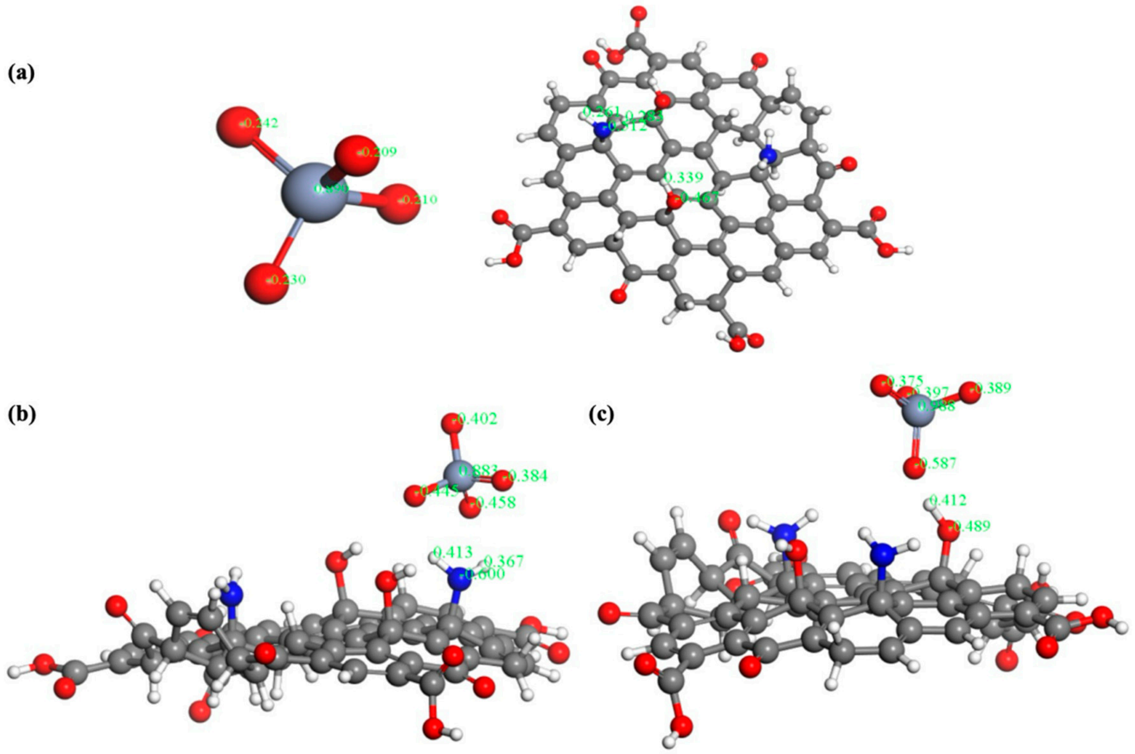

Figure S4. (a) Mulliken charge distribution map of Cr(VI) and N-PCDs. (b) The distribution map of the binding charge of Cr(VI) and N-PCDs at the -NH2 site. (c)The distribution map of the binding charge of Cr(VI) and N-PCDs at the -OH site.
